# Estimating the actual importation risk of dengue virus infection among Japanese travelers

**DOI:** 10.1371/journal.pone.0198734

**Published:** 2018-06-20

**Authors:** Baoyin Yuan, Hiroshi Nishiura

**Affiliations:** Department of Hygiene, Graduate School of Medicine, Hokkaido University, Sapporo, Japan; Institute of Tropical Medicine (NEKKEN), Nagasaki University, JAPAN

## Abstract

**Background:**

Frequent international travel facilitates the global spread of dengue fever. Japan has experienced an increasing number of imported case notifications of dengue virus (DENV) infection, mostly arising from Japanese travelers visiting South and Southeast Asian countries. This has led an autochthonous dengue outbreak in 2014 in Japan. The present study aimed to infer the risk of DENV infection among Japanese travelers to Asian countries, thereby obtaining an actual estimate of the number of DENV infections among travelers.

**Methodology/Principal findings:**

For eight destination countries (Indonesia, Philippines, Thailand, India, Malaysia, Vietnam, Sri Lanka, and Singapore), we collected age-dependent seroepidemiological data. We also retrieved the number of imported cases, who were notified to the Japanese government, as well as the total number of travelers to each destination. Using a mathematical model, we estimated the force of infection in each destination country with seroepidemiological data while jointly inferring the reporting coverage of DENV infections among Japanese travelers from datasets of imported cases and travelers. Assuming that travelers had a risk of infection that was identical to that of the local population during travel, the reporting coverage of dengue appeared to range from 0.6% to 4.3%. The risk of infection per journey ranged from 0.02% to 0.44%.

**Conclusions/Significance:**

We found that the actual number of imported cases of DENV infection among Japanese travelers could be more than 20 times the notified number of imported cases. This finding may be attributed to the substantial proportion of asymptomatic and under-ascertained infections.

## Introduction

Dengue fever is one of the most common mosquito-borne infectious diseases worldwide. The World Health Organization (WHO) estimates that 3.9 billion people in a total of 128 countries are at risk of dengue virus (DENV) infection [1,2]. Of these, more than 100 countries are endemic, extending to the Americas, Eastern Mediterranean, Southeast Asia, and Western Pacific regions [1,2]. *Aedes aegypti* and *Aedes albopictus* are known as the major vector species of DENV. The virus comprises four different serotypes, i.e., DENV-1, DENV-2, DENV-3 and DENV-4, belonging to the genus *Flavivirus*, family *Flaviviridae*. Of the total infections, 80% remain asymptomatic [1,3]. The spectrum of clinical symptoms range from moderate to acute and are sometimes even life-threatening [3,4]. The time from exposure to illness onset (i.e., the incubation period) ranges from 3 to 14 days (most commonly 4–7 days) [5]. Thus, infected travelers can asymptomatically or pre-symptomatically return to Japan from endemic countries without recognizing an infection, thereby potentially increasing the risk of imported dengue outbreaks.

The growing volume of international travel can contribute to the global spread of dengue virus. Japan has experienced an increasing number of notified cases of imported DENV infection, mostly arising from Japanese travelers visiting South and Southeast Asian countries. Such infections have led to an autochthonous dengue outbreak in 2014 in Japan [6]. While the 2014 outbreak was only seasonal (i.e., transmission continued only from August to September), the autochthonous chains of transmission within Japan indicated that the country is at risk of local transmission during the summer season.

A published study analyzed the notification data of imported cases in Japan and calculated the destination country-specific incidence of imported dengue, showing that travelers visiting different Southeast Asian countries have different risks of infection [7]. By exploring not only data of imported cases but also of the volume of travelers, another study demonstrated that the incidence of imported cases reflects the local epidemiological dynamics in dengue-endemic countries [8]. Despite these published studies analyzing imported case data, quantitative risk assessment of DENV infection among susceptible Japanese travelers has yet to be conducted. Owing to a large proportion of asymptomatic infections among individuals infected with DENV, it is possible that a greater number of travelers than reported cases are likely to be exposed to infection without recognition. However, many studies have looked into imported case data alone; as far as we understand, no study has statistically inferred the actual risk of infection among all travelers in endemic countries, thereby estimating the number of DENV infections among travelers. The present study aimed to statistically infer the risk of DENV infection among Japanese travelers to Asian countries to obtain an estimate of the number of DENV infections among travelers.

## Methods

### Data source

We investigated four datasets: (i) notification of imported dengue cases in Japan from 2006 to 2016; (ii) statistics of Japanese international travelers from 2006 to 2016; (iii) age-dependent seroprevalence of DENV in Asian countries; and (iv) average duration of stay among Japanese travelers in each Asian destination country.

Dengue fever is a notifiable disease, according to the Japanese Infectious Disease Control Law. Based on laboratory testing (i.e., virus isolation, polymerase chain reaction, detection of Nonstructural Protein 1(NS1)-antigen, detection of IgM antibody or neutralizing antibody), only confirmed cases are reported [9–11]. Except for a fraction of cases during the 2014 outbreak, all confirmed cases were considered to have acquired infection overseas. We retrieved data of the number of cases and travel history (i.e., destination country) of each imported case from the National Institute of Infectious Diseases [9,10,12]. From 2006 to 2016, dengue infections arose among travelers returning from a total of 63 countries or regions. In the present study, we focused on the following eight countries, which accounted for 83.5% of all notifications during the abovementioned period: Indonesia, Philippines, Thailand, India, Malaysia, Vietnam, Sri Lanka, and Singapore.

We then obtained the volume of Japanese travelers from various sources. According to the National Tourism Organization of Japan [13], the annual number of Japanese travelers was obtained for all eight countries, but only for the period 2011–2015. Traveler was defined as a Japanese having residential address in Japan who entered a foreign country as a visitor. We did not have an access to the information of visit to multiple countries. Additionally, we collected the annual number of Japanese travelers to the Philippines, Thailand, Malaysia, Vietnam, and Singapore from 2006 to 2016 from the Japan Travel Bureau (JTB) Tourism Research & Consulting Co. [14]. For the remaining three countries (especially for datasets from 2006 to 2010 and in 2016), Japanese travelers’ data were retrieved from Statistics Indonesia [15] and the Ministry of Tourism, Indonesia [16], Ministry of Tourism, India [17], and Sri Lanka Tourism Development Authority [18].

Next, we systematically collected seroprevalence data of DENV infection in the abovementioned eight countries from 2006–16. The systematic review was conducted in accordance with the Preferred Reporting Items for Systematic Reviews and Meta-Analyses (PRISMA) statement. We searched MEDLINE and Web of Science databases, using the following search terms:

“Seroprevalence OR Seroepidemic OR Seropositive OR Serological OR Serosurvey OR IgG,

AND dengue OR DENV

AND Indonesia OR Philippines OR Thailand OR India OR Malaysia OR Vietnam OR Sri Lanka OR Singapore”.

**Fig 1:**
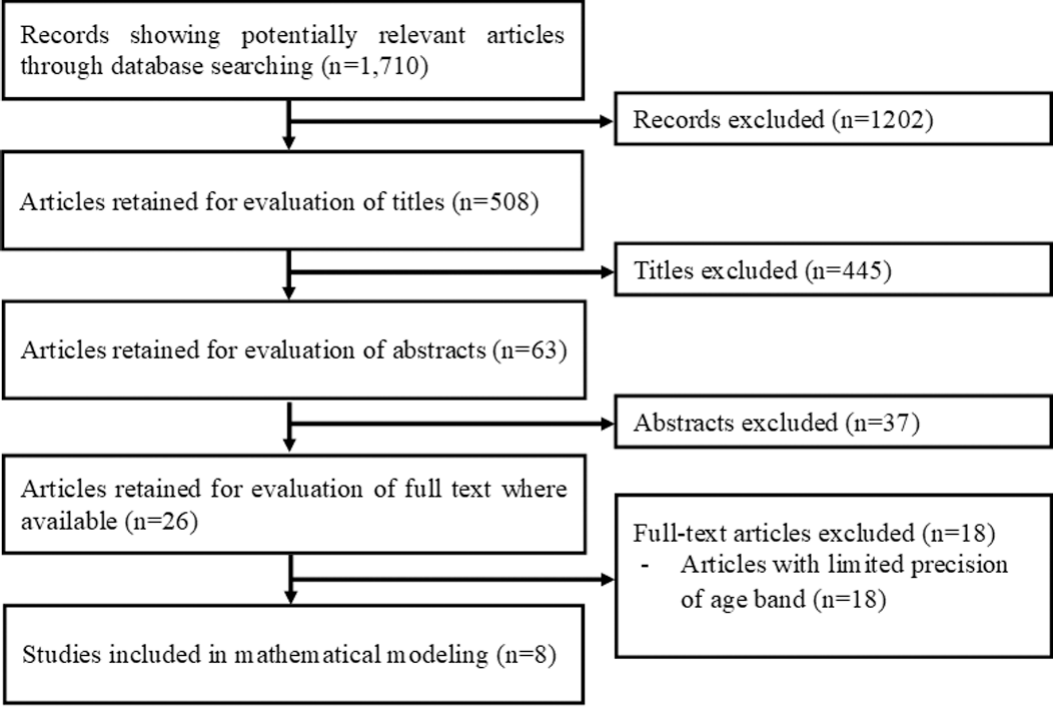
Flow diagram of study selection.

All titles identified by the search strategy were independently screened by two authors (BY and HN). Abstracts of potentially relevant titles were then reviewed for eligibility, and articles were selected for closer evaluation, if a description of seroepidemiological study of dengue was available. Multiple reports of the same country data were assessed, and the latest available data with the best precision of age band was included. Fig 1 shows the flow diagram of study selection. In addition to seroprevalence data, the year of sampling was retrieved; only datasets that were stratified by age group were analyzed. Table 1 shows a summary of seroprevalence data that were reanalyzed in the present study.

**Table 1:** Average length of stay among Japanese travelers and sampling year of seroepidemiological surveys in South and Southeast Asian countries.

Country	Average length of stay [days]^†^	Year of seroprevalence survey	References
Indonesia	8.1	2016	19,25,34
Philippines	9.6	2012	20,26,34
Thailand	6.0	2010	21,27,34
India	10.8	2012	28,33,34
Malaysia	5.9	2008	22,29,34
Vietnam	7.0	2002	24,30,34
Sri Lanka	9.2	2009	18,31,34
Singapore	3.7	2010	23,32,34

^†^The average length of stay that was quoted in each source of information was used.

Finally, the average length of stay per journey to each country was also obtained from multiple data sources. Table 1 summarizes the length of stay data [19–34]. While the year of seroepidemiological study may not be fully consistent with the years of observations of travelers’ dengue, we impose an assumption of stationarity (i.e. the force of infection being a constant) in the mathematical model. Besides, we made the best effort to use the latest available evidence for seroprevalence data so that the mismatch between the time of importation and seroprevalence survey can be minimized.

### Mathematical model

We used a mathematical model to jointly estimate the local hazard (or force) of infection, i.e., the rate at which susceptible individuals experience infection, in each destination country and the coverage of reported DENV infections among Japanese travelers [35]. We used the so-called “catalytic” model in which the fraction of exposed (and immune) individuals is described by the age-independent force of infection *λ* [36, 37]. Assuming that everyone is born susceptible, the expected seropositive fraction at age *a* is

i(a)=1−exp(−λa). (1)

Because dengue is frequently seen in urban settings, we assumed that travelers had a risk (hazard) of infection that was identical to that of the local population. *N*_k_ and *D*_k_ were the annual number of Japanese travelers to country *k* and their average duration of stay, respectively. The expected number of all DENV infections among travelers to country *k* is described as:

$$E\left(i_{k}\right)=N_{k}\left[1-\exp\left(-\lambda_{k}\frac{D_{k}}{365}\right)\right].$$ (2)

Among E(*i*_k_), only the fraction *α*_*k*_ was symptomatic, diagnosed, and notified. Thus, the expected number of imported DENV infections is

$$E\left(c_{k}\right)=\alpha_{k}N_{k}\left[1-\exp\left(-\lambda_{k}\frac{D_{k}}{365}\right)\right].$$ (3)

What Eqs (2) and (3) indicate is that the risk of travelers is assumed as independent of their age. Assuming that the observed number of imported cases follow a Poisson distribution, and also considering that the seroprevalence data is the result of Bernoulli sampling with the expected fraction positive *i*(*a*), the likelihood function to estimate the force of infection and the reporting coverage of DENV infections is

$$L(\lambda_{k},\alpha_{k};A_{k}^{a},B_{k}^{a},c_{k}^{t})\propto \prod_{a}i_{k}{\left(a;\lambda_{k}\right)}^{A_{k}^{a}}{\left(1-i_{k}(a;\lambda_{k})\right)}^{B_{k}^{a}}\prod_{t}\frac{E{\left(c_{k};\lambda_{k},\alpha_{k}\right)}^{c_{k}^{t}}\;\exp(-E(c_{k};\lambda_{k},\alpha_{k}))}{c_{k}^{t}!}$$ (4)

where *A*^*a*^ and *B*^*a*^ are the number of seropositive and seronegative individuals at median sampling age *a*, respectively. Maximum likelihood estimates of unknown parameters were obtained by minimizing the negative logarithm of Eq (4). We calculated 95% confidence intervals using the profile likelihood method.

Travelers may experience a hazard of infection that differs from that of the local population. As part of sensitivity analysis, we explored the impact of having a different force of infection using the relative hazard *ε*, i.e.,

$$E\left(c_{k}\right)=\alpha_{k}N_{k}\left[1-\exp\left(-\varepsilon \lambda_{k}\frac{D_{k}}{365}\right)\right].$$ (5)

Using the observed number of imported cases, yearly number of travelers, and estimated reporting coverage, the risk of infection among travelers to destination *k* in year *t* was calculated as

$$p_{k}^{t}=\frac{c_{k}^{t}}{\alpha_{k}N_{k}},$$ (6)

per journey. To jointly estimate the force of infection and reporting coverage, the following assumptions were made.

1. International travelers have a hazard of infection that is identical to that of the local population in each dengue-endemic country.
2. Two model parameters *α* and *λ* were assumed to be constant over time. Namely, dengue was approximately in a stationary state.
3. Seasonality was discarded owing to a shortage of finer scale data of travelers.

### Ethical considerations

The present study analyzed only secondary datasets that were publicly available, all of which were de-identified before collection. As such, ethical approval by the Institutional Review Board was not required for the present study.

### Availability of supporting data

The dataset of imported dengue cases used in the present study is available as Online Supplementary Material.

## Results

**Fig 2:**
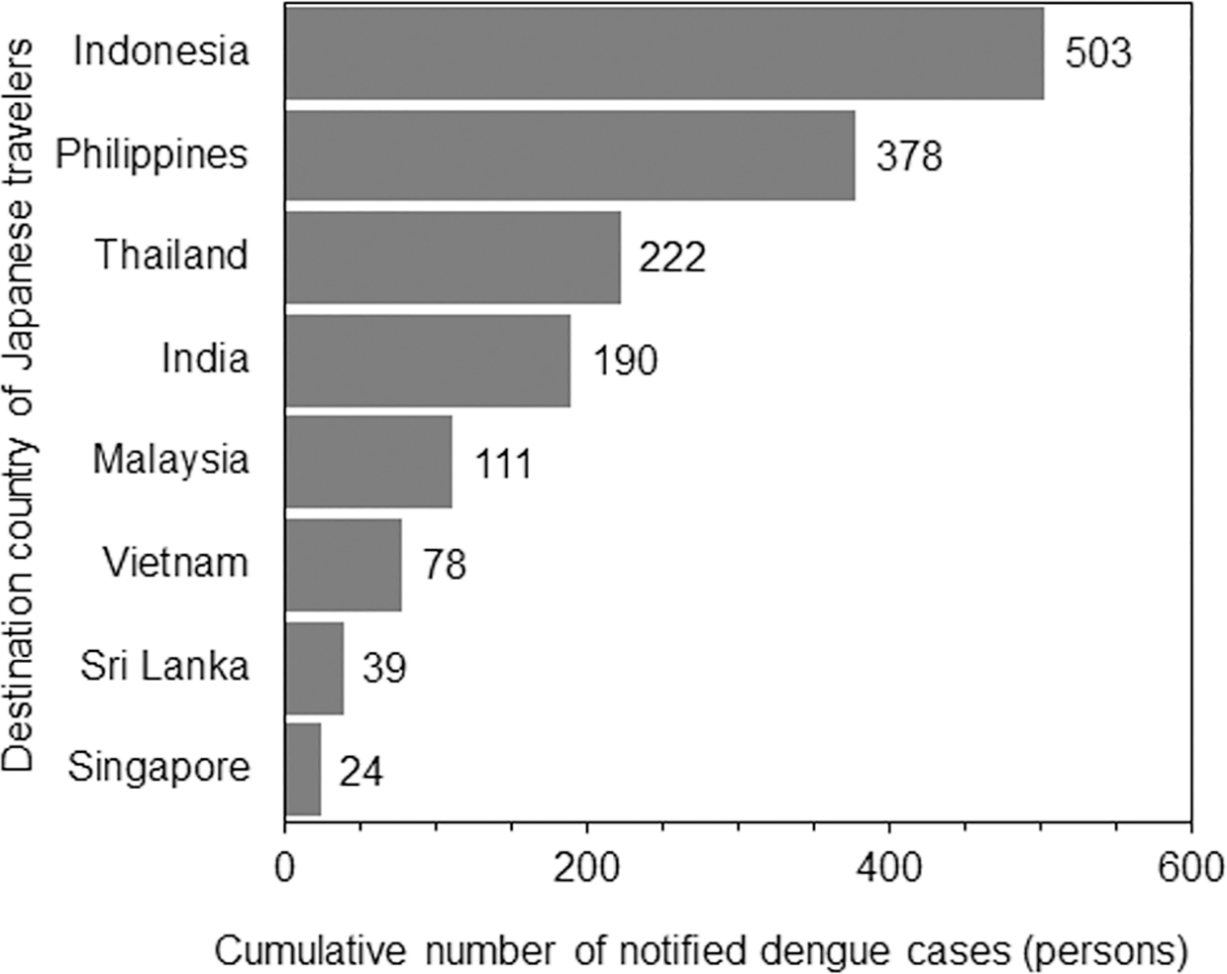
Cumulative number of imported cases of dengue virus infection from 2006 to 2016 by destination country. Eight countries are shown in descending order of cumulative number of cases. Only notified cases based on laboratory diagnosis are counted.

From 2006 to 2016, there were a total of 2026 imported cases of DENV infection from 63 different countries that could potentially act as the source of exportation (Online Supplementary Material). Of these, 176 patients visited multiple countries and their place of infection was not determined. Of the remainder (1850 patients), South and Southeast Asian countries accounted for the majority of cases. In descending order of incidence, imported cases came from Indonesia, Philippines, Thailand, India, Malaysia, Vietnam, Cambodia, Myanmar, Sri Lanka, Bangladesh, and Singapore. Owing to unavailability of seroepidemiological survey data, Cambodia, Myanmar, and Bangladesh were excluded and the remaining eight countries were included for further analyses (Fig 2). Imported cases from the eight selected countries accounted for 83.5% of the total notifications.

**Fig 3:**
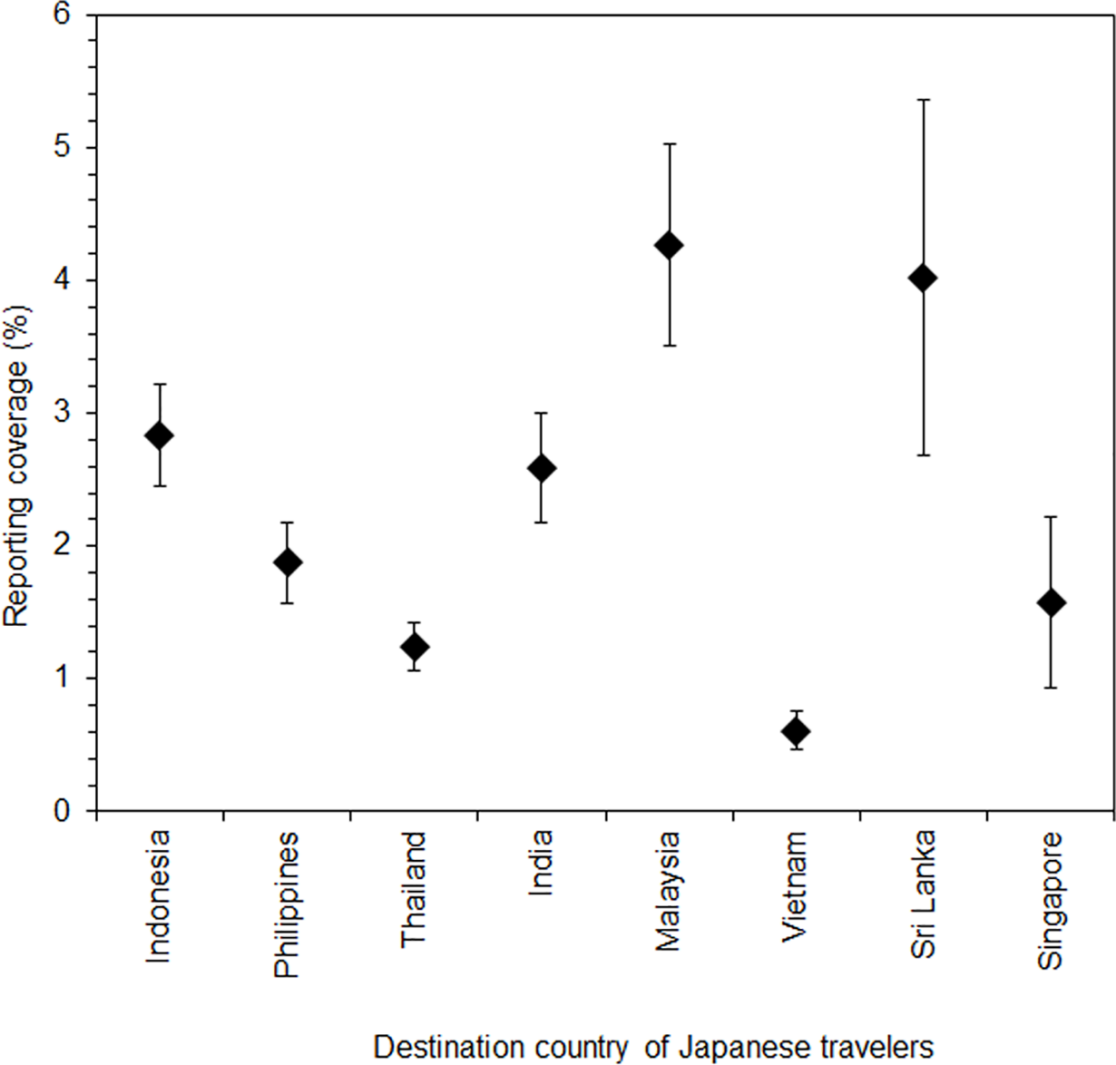
Estimated reporting coverage of dengue virus infection among travelers by destination country. Filled diamonds represent maximum likelihood estimates; whiskers extend to lower and upper 95% confidence intervals. Travelers were assumed to have experienced an identical force of infection of dengue virus infection to the local population.

Table 2 shows the maximum likelihood estimate of the force of infection in each country. High estimates of the force of infection were obtained for the Philippines, Sri Lanka, and Indonesia followed by Vietnam and India. Assuming that travelers experienced an identical hazard of dengue infection during their journey, the reporting coverage *α* was estimated for each country (Fig 3). The reporting coverage of dengue ranged from 0.6% to 4.3%, implying that the actual number of DENV infections is 23.3–166.7 times greater than the reported number of cases. Malaysia and Sri Lanka had the highest estimate of reporting whereas Vietnam yielded the lowest value.

**Table 2:** Estimated force of infection of dengue virus infection in South and Southeast Asian countries.

Country	Force of infection, per year
Indonesia	0.15 (0.14, 0.17)
Philippines	0.17 (0.15, 0.19)
Thailand	0.08 (0.07, 0.08)
India	0.13 (0.12, 0.13)
Malaysia	0.05 (0.05, 0.05)
Vietnam	0.12 (0.11, 0.13)
Sri Lanka	0.14 (0.13, 0.15)
Singapore	0.02 (0.02, 0.02)

Force of infection in parenthesis shows 95% confidence intervals derived using the profile likelihood method.

The impact of seasonal force of infection on the estimate of *α* can be explored using Eq (3). In our analysis, given observed number of imported cases *c* and travelers *N*, we have used the following equation:

$$\alpha_{0}=\frac{c}{N\left[1-\exp\left(-\lambda_{0}d\right)\right]},$$ (7)

where *d* is the length of stay (years). However, it is possible that we missed the seasonal force of infection which may be *h* times greater than our assumed constant, i.e., *λ* = *hλ*_0_ where *h*>1.0 during the summer season when the mosquito vector is active. Then the actual *α* that we should have estimated would be

$$\alpha =\frac{c}{N\left[1-\exp\left(-h\lambda_{0}d\right)\right]},$$ (8)

yielding the extent of bias by ignoring the seasonality as

$$\frac{\alpha }{\alpha_{0}}=\frac{1-\exp\left(-\lambda_{0}d\right)}{1-\exp\left(-h\lambda_{0}d\right)}\approx \frac{1}{h}.$$ (9)

That is, if the force of infection during the summer is *h* times greater than the assumed average, the estimated *α* was biased by 1/*h* times.

**Fig 4:**
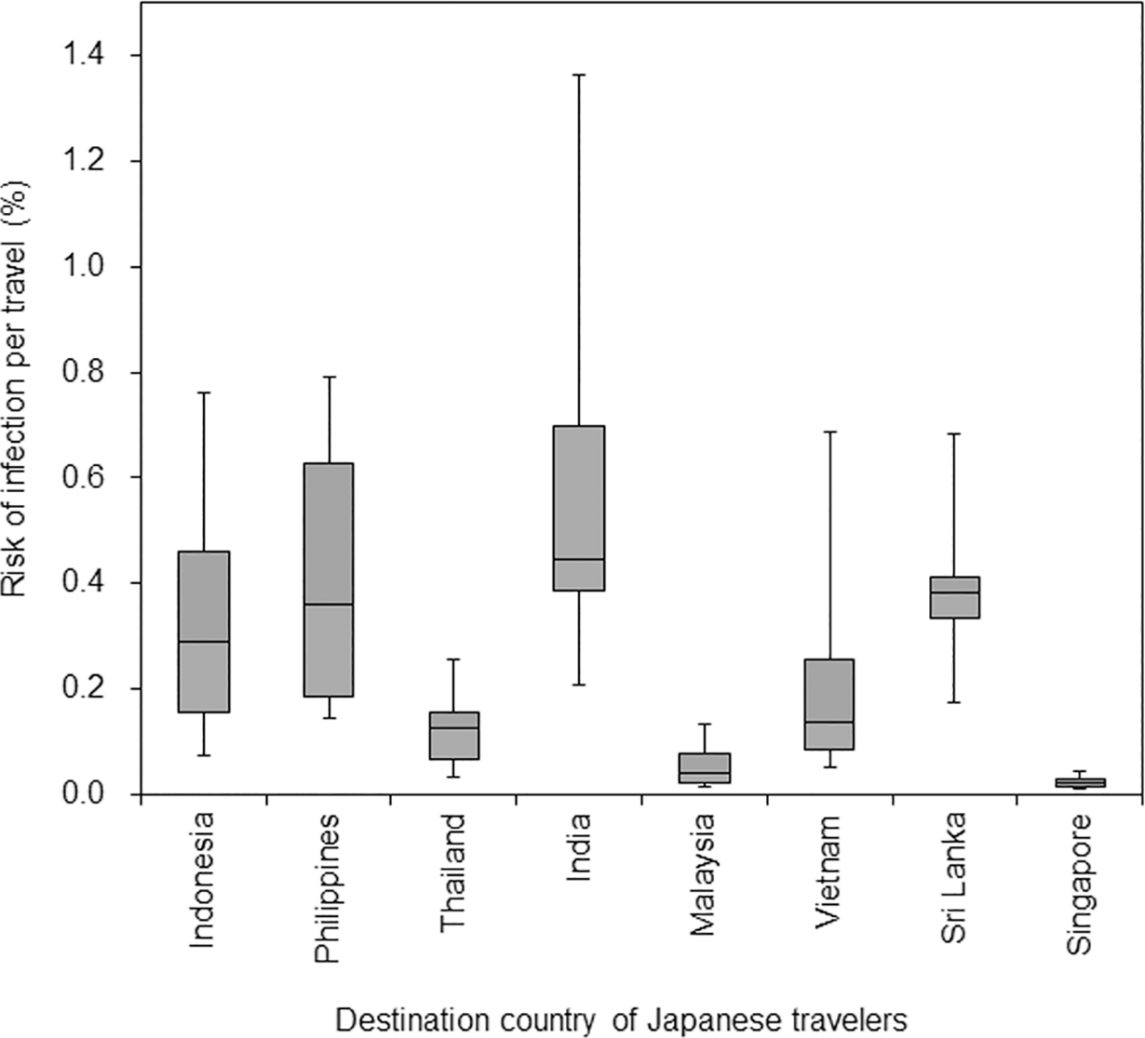
Estimated risk of dengue virus infection among Japanese travelers from 2006 to 2016 by destination country. Box whisker plot shows the range of estimated risk of infection per journey from 2006 to 2016. Middle line of box represents median, extending from lower to upper quartiles. Whiskers extend to lowest and highest risks.

Using the observed number of imported cases, yearly number of travelers, and estimated reporting coverage, the risk of infection among travelers to destination *k* was calculated. The variation of estimated risk from 2006 to 2016 is shown in Fig 4. The risk of infection per travel ranged from 0.02% to 0.44%. The lowest estimate was obtained in Singapore and the highest from India.

**Fig 5:**
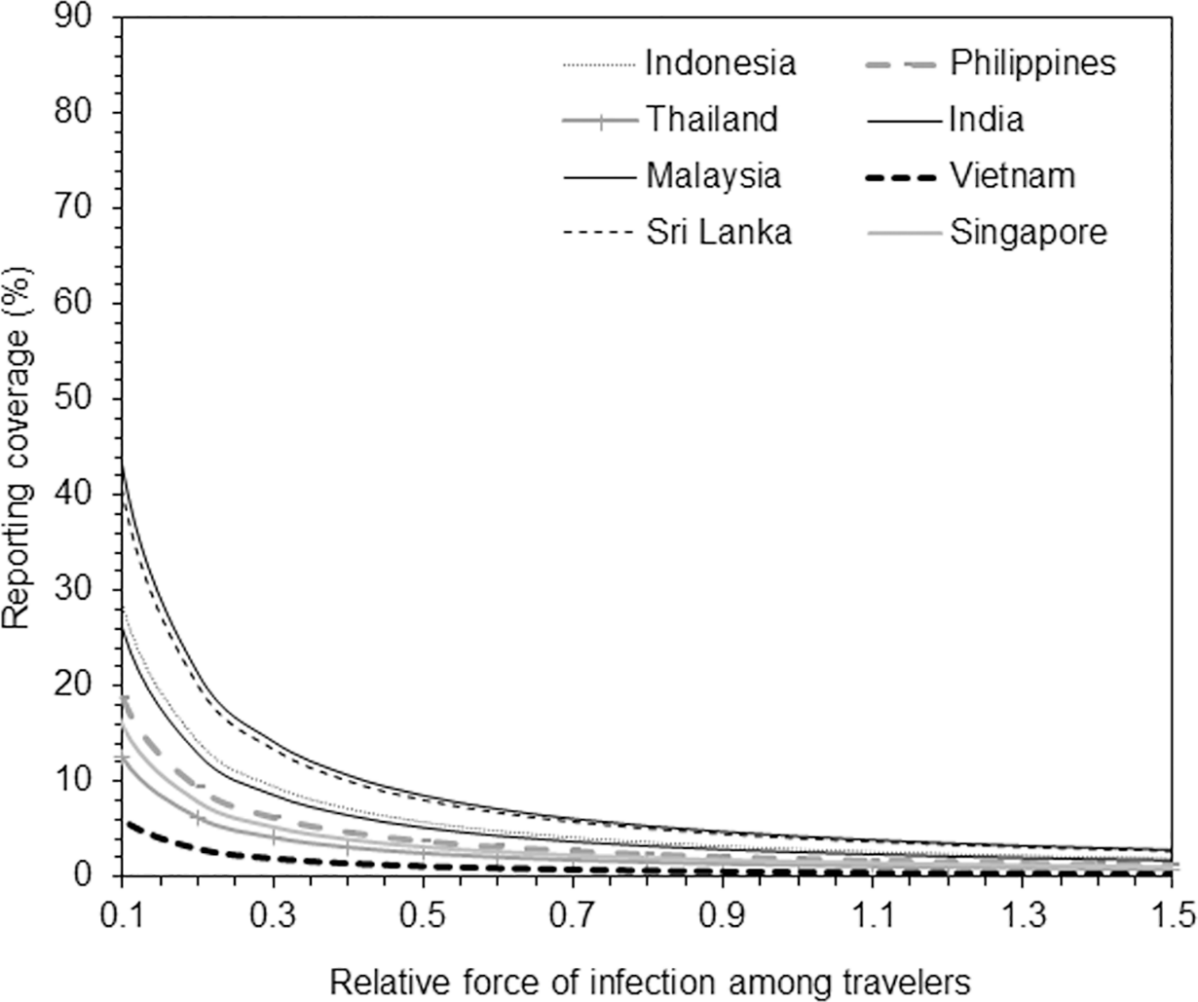
Sensitivity of reporting coverage to the relative hazard of travelers compared with local population. Relative hazard on horizontal axis shows the relative hazard, obtained as the ratio of the force of infection among Japanese travelers to that of the local population in each destination country. Vertical axis measures the estimated reporting coverage; only maximum likelihood estimates are shown.

Because travelers could have risk of infection that differed from that of the local population, we examined the sensitivity of reporting coverage to relative hazard *ε*. Fig 5 shows that the reporting coverage would be considerably increased if the hazard were lower than 1. Nevertheless, a dramatic increase of reporting coverage was seen only when *ε* was substantially lower than 1 (e.g., lower than 0.5).

## Discussion

In the present study, we statistically inferred the risk of DENV infection among Japanese travelers to Asian countries, as the importation is recognized as increasing over time [38]. To accomplish this task, we used multiple pieces of data as a trick to estimate asymptomatically infected fraction, including seroprevalence data of travelers’ destination country such that the force of infection could be explicitly estimated. Integrating the force of infection into the process of importation and assuming that travelers experienced a force of infection identical to that of local people during their travel, the reporting coverage of dengue was estimated to range from 0.6% to 4.3%. This finding implies that the actual importation of DENV infection among Japanese travelers may be more than 20 times the notified number of imported cases. Moreover, the risk of infection per travel ranged from 0.02% to 0.44%.

There are two take home messages from the results of our study. First, our modeling exercise allowed us to demonstrate that the observed number of imported cases truly represents the tip of the iceberg. Given that the Japanese government is annually notified of 200 imported cases per year [8,39,40], one would expect that 4,000 (or even more) imported DENV infections could have occurred; however, the majority of infections remain unrecognized. While the finding is subject to validation through seroprevalence survey among all travelers, including healthy individuals, coming back from Southeast Asia, the present study is the first to explicitly show how DENV infections are under-ascertained among Japanese travelers who are asymptomatic or mildly infected.

Second, the risk of infection among travelers was explicitly estimated to range from 0.02% to 0.44% per journey. These figures indicate that there have been an estimated 20–440 DENV infections per 100,000 travelers visiting South and Southeast Asian countries. Of course, this estimated risk of infection was greater than the published estimate [8], which depends on reported cases alone. It is critical to consider the undetected proportion of infected individuals when determining the risk of local outbreaks in Japan because these individuals can contribute to secondary transmission despite being asymptomatic or undiagnosed [41]. For instance, undiagnosed individuals might be bitten by *Aedes* species during their infectious period while being unaware of their own risk of causing a dengue outbreak [42,43].

Three technical problems in this study must be noted. First, we ignored spatial heterogeneity. The seroprevalence data that we analyzed could have relied on observations in a particular geographic locality, and representativeness of the sample may not be fully guaranteed. The risk of dengue would undoubtedly vary with geographic space [44], but travelers’ data were only classified by the destination country to which they traveled. In the future, precise estimation of the risk of dengue among travelers should ideally rest on more detailed information with respect to travel patterns (e.g. volume and risky behavior of travelers by different destinations) and seroprevalence data in each geographic locality.

Second, the present study ignored more precise temporal resolution. Monthly dengue incidence data were unavailable for all eight destination countries, and we had to compromise by using yearly numbers of cases as well as travelers. Not only dengue transmission but also tourism pattern involves seasonality, but we had to ignore this aspect, which we aim to improve in the future study. At least, we have mathematically clarified the impact of seasonal force of infection on the estimate of ascertainment probability. Moreover, it should be noted that there was a mismatch of timing in the datasets, e.g., Vietnam in 2002 and 2006–2016 for seroepidemiological and case data, respectively. Whereas estimates in Vietnam did not appear to be considerably different from others, such mismatch should be filled by updated seroepidemiological data in the future.

Third, we made an inherent assumption that the force of infection among travelers was identical to that of the local population during travel; we were unable to fully validate this assumption. Especially, it is critical to focus on travelers’ behavior during the stay: the extent of risky behaviors would greatly differ by the purpose of visit. We believe that the assumption of identical risk was qualitatively sound because most travelers’ tourist destinations were capital cities (e.g., Bangkok); the main spatial hotspot of a dengue epidemic is frequently found in urban settings. While the issue of spatial representativeness still remains (e.g. mismatch between travelers’ destination and seroprevalence survey area; Binh Thuan province in Vietnam), we believe that the assumption holds better for dengue with urban foci than malaria which requires biting events during night time in rural area. At the very least, it should be remembered that the extent of underestimation that we have shown might have been overemphasized if the force of infection among travelers was lower than that of the local population.

While all these limitations cannot be immediately overcome, we believe that we have successfully quantified the reporting coverage of DENV infection among travelers returning to Japan, yielding the estimate of the risk of infection per travel. Evaluation relying only on reported cases can result in remarkable underestimation of the risk of infection. The existence of a substantial number of unrecognized infections could trigger local outbreaks of dengue in Japan; therefore, more precise data collection and monitoring of travelers is required.

## Supporting information

S1 Table Annual number of imported cases of dengue virus infection in Japan from 2006 to 2016. (XLSX)
